# Electrochemical and Spectroscopic (FTIR) Evidence of Conducting Polymer-Cu Ions Interaction

**DOI:** 10.3390/molecules28020569

**Published:** 2023-01-06

**Authors:** Gerardo Salinas, Bernardo A. Frontana-Uribe

**Affiliations:** 1 Departamento de Química Orgánica, Centro Conjunto de Investigación en Química Sustentable UAEM-UNAM, Km 14.5 Carretera Toluca-Atlacomulco, Toluca 50200, Mexico; 2 Departamento de Química Orgánica, Instituto de Química, Universidad Nacional Autónoma de México, Ciudad Universitaria, Ciudad de México 04510, Mexico

**Keywords:** 3,4-alcoxy-thiophenes, Fourier-transform infrared spectroscopy, square wave voltammetry, cyclic voltammetry, stripping voltammetry, polymer-ions interaction

## Abstract

In this work, we provide electrochemical and spectroscopic evidence of the conducting polymer-heavy metal ion interaction by comparing the electrochemical and spectroscopic behavior (FTIR) of two different conducting polymer-modified electrodes based on 3,4-alkoxythiophenes: 3,4-ethylenedioxythiophene (EDOT) and *ortho*-xylen-3,4-dioxythiophene (XDOT) during the potentiodynamic stripping of copper. By analyzing the electrochemical and spectroscopic results, it is possible to propose two different copper dissolution processes during the electrochemical stripping process, which depend on the conducting polymer used. With PEDOT matrix, stripping occurs in a two-step pathway, observed as two anodic peaks, involving the formation of the Cu^+^-PEDOT complex and the subsequent oxidation step of the Cu^+^ complex to release Cu^2+^ ions. On the other side, the experiments carried out let us propose the formation of a poorly stable Cu^2+^-PXDOT complex or a superficial mechanism for the Cu^2+^ release, characterized by a single stripping signal for this process. Thus, the incorporation of Cu ions into the matrix and the stripping release are intimately related to the chemical structure of the polymer used.

## 1. Introduction

Chemically modified electrodes (CME) have gained considerable attention due to their different applications ranging from electroanalysis to energy conversion [1,2,3]. Among the different types of CME, electrodes modified with conducting polymers present considerable advantages due to their possible use as ion exchange membranes and chemical or biochemical sensors [4,5,6,7]. This is attributed to their intrinsic high surface area and porosity, in synergy with the possible interaction between the electron withdrawing or donating groups contained in the polymer structure, and the analyte of interest [8]. In this frame, it could be possible to take advantage of the chemical interactions between the electron-rich atoms with available electron pairs such as N, O, and S within the polymeric structure and heavy metal ions, to enhance the sensitivity and selectivity toward these ions. In addition, the presence of metal ions in the polymeric matrix improves the conductivity and crystallinity of the material [9,10]. Thus, such hybrid surfaces have gained attention in different fields such as electrocatalysis, microelectronics, and corrosion.

Commonly, such metallic/polymeric composites can be obtained by metallic sputtering, electroless, or conventional electrochemical deposition. It is well-established that by using electrochemical methods, it is possible to stabilize metal ions in different redox states in the polymer matrix. Such an effect has been proposed for different metallic ion/polymer systems. Such as Cu in polyaniline [11], Ag, Au, and Cu in polypyrrole [12,13,14,15,16,17], Cu in poly-3-methylthiophene [18], and Cu, Pd, and Ag in poly-3,4-ethylenedioxythiophene (PEDOT) [19,20,21,22,23,24]. In all these examples, an interaction between the partially reduced metal and the nitrogen or sulfur atoms within the polymer matrix is proposed, which results in a so-called metal/polymer complex. Ilieva et al. proposed a mechanism for the formation of Cu-PEDOT complexes, in which copper atoms are oxidized to Cu^+^, stabilized by the polymeric matrix [19,20]. Furthermore, it has been reported that the mechanism for the recognition of Cu^2+^ using electrodes modified with PEDOT consists of two steps: (i) an oligomeric reorganization, and (ii) the copper nucleation [25].

**Scheme 1:**
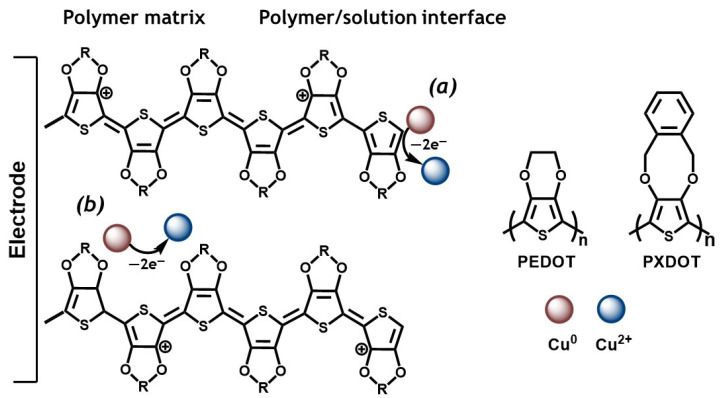
Schematic illustration of the mechanism of recognition of copper using electrodes modified with alkoxythiophenes and the chemical structures of 3,4-ethylenedioxythiophene (EDOT) and *ortho*-xylen-3,4-dioxythiophene (XDOT).

In this work, we provide electrochemical and spectroscopic (FTIR) evidence of the conducting polymer-heavy metal ion interaction by comparing the electrochemical and spectroscopic behavior of two different conducting polymer-modified electrodes based on 3,4-alkoxy thiophenes: 3,4-ethylenedioxythiophene (EDOT) and *ortho*-xylen-3,4-dioxythiophene (XDOT) (Scheme 1). The latter has gained our attention due to its outstanding electrochemical, electrical, and optical stability, even in hydroalcoholic media [26,27,28]. Recently, the possible quantification of Cu^2+^ through the use of modified electrodes with PXDOT was studied [29]. However, a better understanding of the possible interaction between the matrix of these π-conjugated polymers and heavy metal ions is still needed and is the focus of this contribution.

## 2. Results

**Figure 1:**
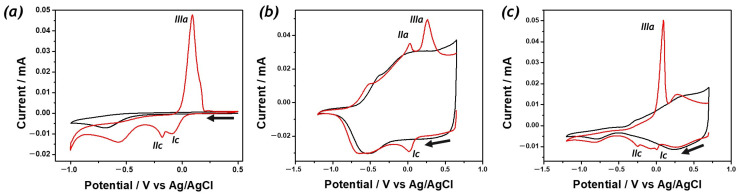
Potentiodynamic reduction of a 0.1 M LiClO_4_, acetate buffer (pH 4) aqueous solution with 100 ppm Cu^2+^ (red line) and without Cu^2+^ (black line) using (**a**) GC, (**b**) PEDOT, and (**c**) PXDOT electrodes.

As stated above, the oxidation of copper using a PEDOT-modified electrode takes place at either a) the polymer/solution interface, or b) at the polymer matrix (Scheme 1) [19,20]. Thus, Cu^+^- and Cu^2+^-PEDOT complexes stabilized by the electron-rich groups within the alkoxy thiophene have been proposed. In order to corroborate this, at first the potentiodynamic stripping of copper was evaluated at a bare glassy carbon electrode (GC) (Figure 1a). Under this condition, three different redox processes associated to the copper stripping can be observed: the reduction of Cu^2+^ and Cu_2_O to Cu^0^ (peak Ic, *E*_(*Ic*)_ ≈ −0.09 V vs. Ag/AgCl and peak IIc, *E*_(*IIc*)_ ≈ −0.18 V vs. Ag/AgCl, respectively) and the oxidation of Cu^0^ to Cu^2+^ (peak IIIa, *E*_(*IIIa*)_ ≈ 0.09 V vs. Ag/AgCl) [30,31]. However, the potentiodynamic stripping of copper on the surface of the alkoxy thiophenes presents considerable differences in comparison with the bare GC. For example, on the PEDOT-modified electrode, one cathodic peak (peak Ic, *E*_(*Ic*)_ ≈ 0.02 V vs. Ag/AgCl), and two anodic signals (peak IIa, *E*_(*IIa*)_ ≈ 0.03 V vs. Ag/AgCl and peak IIIa, *E*_(*IIIa*)_ ≈ 0.27 V vs. Ag/AgCl, respectively) were obtained (Figure 1b). Whereas the PXDOT electrode presented two reduction processes (peak Ic, *E*_(*Ic*)_ ≈ 0.01 V vs. Ag/AgCl and peak IIc, *E*_(*IIc*)_ ≈ −0.25 V vs. Ag/AgCl, respectively) and one anodic process (peak IIIa, *E*_(*IIIa*)_ ≈ 0.09 V vs. Ag/AgCl) (Figure 1c). It is important to highlight that the cathodic peak between −0.7 V and −0.5 V vs. Ag/AgCl is attributed to the reduction of oxygen; however, due to the controlled pH of the working solution, the formation of the copper hydroxide complex is minimized.

**Figure 2:**
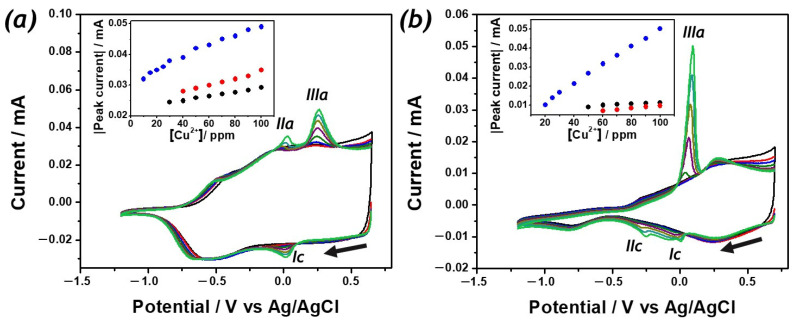
Potentiodynamic reduction at (**a**) PEDOT and (**b**) PXDOT electrodes of a 0.1 M LiClO_4_, acetate buffer (pH 4) aqueous solution at different Cu^2+^ concentrations. Inset shows a plot of the peak current (absolute value) vs. the concentration of added copper for peak Ic (black dots), IIa (red dots), and IIIa (blue dots).

To provide a deeper insight into the electrochemical behavior of the copper stripping on the alkoxy thiophenes, different concentrations of the metallic ion were studied (Figure 2). For both electrodes, the signals were concentration-dependent. Furthermore, in the case of PEDOT, peak IIIa appears at a relatively low copper concentration, whereas signals Ic and IIa are visible at Cu^2+^ concentrations above 30 ppm (Figure 2a). From these potentiodynamic plots, it is relatively straightforward to assume that the redox processes take place through the formation of a metallic complex intermediary, as established by Ilieva et al. [19,20]. However, these results only allow us to associate the peak Ic with the direct reduction of Cu^2+^ to Cu^0^, whereas the oxidation, according to previous reports, follows a possible two-reaction pathway from Cu^0^ to Cu^+^, stabilized by the formation of a Cu^+^-PEDOT complex (peak IIa), and from Cu^+^ to Cu^2+^ (peak IIIa) [19,20]. Nonetheless, since the redox processes Ic and IIa begin at high copper concentrations in comparison to IIIa, it is possible to conclude that such reactions take place exclusively in the polymer matrix. By using the PXDOT electrode, the peak IIIa appears at a low copper concentration, whereas the signals Ic and IIc are visible at Cu^2+^ concentrations above 50 ppm (Figure 2b); thus, a different mechanistic pathway can be proposed. In this case, the peak IIIa can be associated with the direct oxidation of Cu^0^ to Cu^2+^, whereas the cathodic sweep involves the reduction of Cu^2+^ to Cu^0^ (peak Ic) and from Cu_2_O to Cu^0^ (peak IIc). The absence of a stripping peak associated with the oxidation of Cu^0^ to a Cu^+^-PXDOT complex enables us to assume that such an intermediate state of Cu is unstable in the experimental conditions. This is attributed to a different stabilization effect compared with PEDOT, where it is more efficient compared with PXDOT, as has been observed with other Cu(I) complexes [19,20].

**Figure 3:**
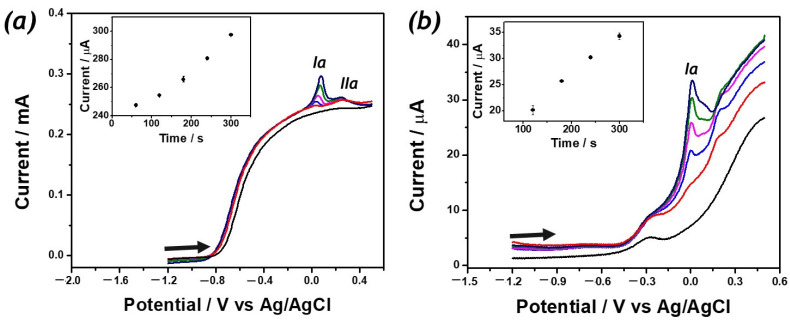
SWASV at different deposit times (from 60 to 300 s) in a 0.1 M LiClO_4_ acetate buffer (pH 4), obtained with a (**a**) PEDOT and (**b**) PXDOT electrode in the presence of 2 ppm and 0.1 ppm of Cu^2+^, respectively. For all the experiments, *E_d_* = −1.0 V, *f* = 15 Hz, *Δes* = 4 mV, *E_SW_* = 50 mV as analytical parameters. The accumulation of the metal ion was carried out under stirring. Inset shows a plot of peak current vs. the deposit time.

After this set of experiments, we evaluated if the redox reactions took place either at the polymer/solution interface or at the polymer matrix, as depicted in the Scheme 1. This was carried out using square wave anodic stripping voltammetry (SWASV), which enables an efficient elimination of the capacitive current with enhanced sensitivity [32,33,34]. The SWASV of 0.5 ppm of Cu^2+^ on a GC electrode showed the characteristic stripping peak of copper at −0.03 V vs. Ag/AgCl and the oxygen reduction peak at −0.57 V vs. Ag/AgCl (peak Ia and IIa, respectively, Figure S1). The stripping process of copper at the surface of the alkoxy thiophene-modified electrodes was evaluated by changing the deposit time from 60 to 300 s. As it can be seen in Figure 3a, by using the PEDOT-modified electrode, the copper stripping follows the above mentioned two-redox-reaction system. However, the current intensity of the peak Ia increases as a function of the deposit time (Figure 3a), whereas the current of the Iia remains partially constant. Castillo-Lara et al. demonstrated that the stripping mechanism of copper on PEDOT involves a reorganization of the oligomeric chains followed by the copper nucleation [25]. In addition, these processes take place simultaneously in the oligomeric matrix and on the PEDOT/solution interface. Since during the accumulation step PEDOT is in its insulating state, the reduction of copper takes place exclusively in the vicinity of the GC surface, either by an inner or outer sphere electron transfer mechanism. Furthermore, the amount of Cu^2+^ ions that can diffuse inside the polymer matrix is time-dependent. Thus, it is possible to conclude that under these conditions, the redox process associated with peak Ia occurs at the polymer matrix, whereas peak IIa is limited to the polymer/solution interface. In the case of the PXDOT-modified electrode, only one stripping peak was obtained, and its current intensity is time-dependent (Figure 3b). Thus, the direct oxidation of Cu^0^ to Cu^2+^ takes place mainly at the PXDOT polymer surface, limiting the metal–polymer interactions that stabilize Cu^+^.

Finally, to provide more evidence of the formation of the metallic complex within the polymeric matrix, a Fourier-transform infrared spectroscopy (FTIR) was carried out on the obtained polymer films. At first, the polymers were deposited potentiodynamically on the surface of a flexible Au-coated carbon sheet (Figure S2). Afterwards, two different sets of samples were analyzed separately by FTIR: (1) modified electrodes dipped for 24 h in a 0.1 M CuSO_4_ solution in order to evaluate the possible interaction between free Cu^2+^ ions and the poly-3,4-alkoxythiophenes, and (2) electrodes for the potentiostatic copper deposition/dissolution process, performed in a 1000 ppm Cu^2+^ solution, aimed to provide evidence of the formation of metal ion complexes at different potentials.

The FTIR spectra of the pristine polymers present two characteristic vibration bands associated with the carbon–carbon double bond at around 1280 and 1450 cm^−1^ (Figure S3, black line and blue region), and signals of around 1040 and 1170 cm^−1^ corresponding to the ether groups (Figure S3, black line and green region). After 24 h in a 0.1 M CuSO_4_ solution, for PEDOT, the bands around 1600 and 1000 cm^−1^ decrease by more than 50% (Figure S3a green line), whereas for PXDOT, only the signal around 1170 cm^−1^ decreases by ca. 30% (Figure S3b, green line). These results are evidence of the existence of different interactions between the alkoxy groups within the polymer structure and the Cu^2+^ ions. 

**Figure 4:**
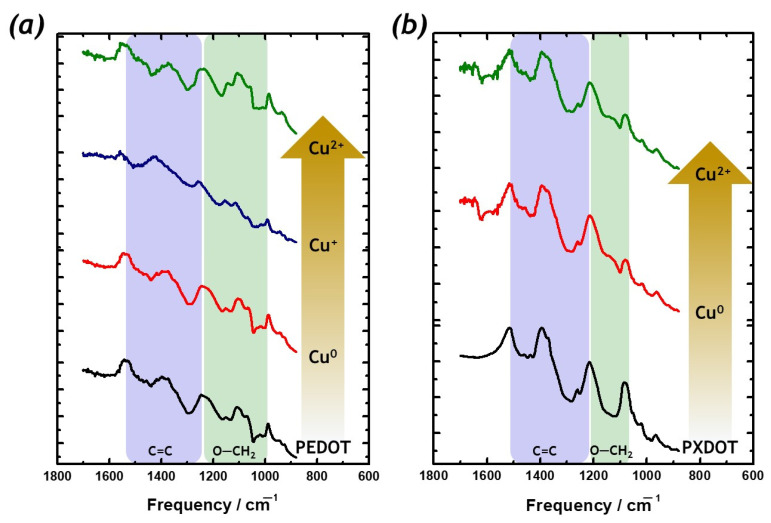
FTIR spectra of (**a**) PEDOT and (**b**) PXDOT films obtained before (black line) and after potentiostatic modification at different pulse potentials: −0.4 V (red line), 0.15 V (blue line), and 0.4 V (green line). The accumulation of the metal ion was carried out under stirring for 120 s.

The FTIR spectra of the polymer films after electrochemical modification at different pulse potentials corroborated these findings. The spectra obtained at −0.4 V vs. Ag/AgCl for PEDOT present no significant changes (Figure 4a, red line), whereas for PXDOT only, a slight decrease of the vibration band around 1170 cm^−1^ was obtained (Figure 4b, red line). After a pulse potential of 0.15 V vs. Ag/AgCl, above the first copper stripping peak on PEDOT, the spectrum presents the complete depletion of all the vibration bands (Figure 4a, blue line). Finally, the FTIR spectra obtained after applying a potential of 0.4 V vs. Ag/AgCl, present a partial recovery of all the vibration bands for PEDOT (Figure 4a, green line). This behavior agrees with the formation of a Cu^+^-PEDOT and a Cu^2+^-PEDOT proposed by Ilieva et al. [19,20]. In the case of PXDOT, the vibration bands associated with the carbon–carbon double bond and the one corresponding to the ether group decrease around 50% and 70% respectively, but never disappear (Figure 4b, green line), which is explained as a more superficial deposit with fewer metal–polymer interactions.

## 3. Discussion

After analyzing the electrochemical and spectroscopic results, it is possible to propose two different copper dissolution processes during the electrochemical stripping process. First, the reduction of copper takes place at the interface of the electrode, and then the copper ions diffuse differently toward the polymer matrix depending on the polymer used. The presence of the deposited metal only affects the vibration bands of the ether group of the PXDOT film. Afterwards, the anodic stripping of copper at the PEDOT matrix occurs in a two-step pathway involving the formation of the Cu^+^-PEDOT complex, which was corroborated by the FTIR due to the complete depletion of all the characteristic vibration bands. In the first oxidation step, the Cu^+^ complex is oxidized by the Cu^2+^-PEDOT, which takes place exclusively in the polymer matrix. It is important to highlight that the anodic current associated with the oxidation of Cu^0^ to the Cu^+^-PEDOT complex probably overlaps with the current associated with the charging of PEDOT, which limits its visualization. For the second oxidation process, the Cu^+^-PEDOT complex oxidizes to a free Cu^2+^ ion on the polymer/solution interface, which produces the complete recovery of the initial FTIR spectra. According to the FTIR spectra, the Cu^+^-PEDOT complex is formed by the interaction between the carbon–carbon double bonds, as well as the ether moiety of the 3,4-ethylenedioxythiophene ring. The electrochemical behavior, in synergy with the spectroscopic results, allows us to propose the formation of a poorly stable Cu^2+^-PXDOT complex with a superficial mechanism for the Cu^2+^ release, showing a single signal for this process. In addition, the slight change in the vibration bands associated with the carbon–carbon double bond and the ether group corroborates this proposal for the PXDOT matrix. In this way, the reduction and oxidation of Cu^2+^ depend on the polymeric matrix used.

The results presented in this work are the first pieces of evidence of the chemical interaction between electron-rich atoms within different conducting polymer-modified electrodes based on 3,4-alkoxy thiophenes and a heavy metal ion. In particular, such interaction with copper ions is of outstanding importance in electroanalysis. It is well-known that high concentrations of this metal ion can cause serious damage to the liver and brain [35,36]; therefore, its efficient quantification is needed. Thus, it is possible to take advantage of the understanding of such interactions to enhance the sensitivity and selectivity toward heavy metal analysis, using modified electrodes with conducting polymers based on alkoxy thiophenes [5,37]. Finally, the authors envisage similar interactions between alternative heavy metals such as Pb^2+^, Cd^2+^, or Zn^2+^ and the electron-rich groups within the alkoxy thiophene conducting polymers, which will lead to a better understanding of the metal–polymer interactions that occur with these environmentally important metallic ions. This will open the use of this family of π-conjugated polymers for electroanalysis in complex liquid matrices.

## 4. Materials and Methods

LiClO_4_ (Aldrich (St. Louis, MI, USA), 99.9%), glacial acetic acid (Aldrich, 99.7%), sodium acetate (Acros Organics, Geel, Belgium, 99%), and 3,4-ethylenedioxythiophene (Aldrich, 97%) were used as received. The Cu standard stock solution (1000 ppm) was prepared by dissolving pure metal commercial wires in nitric acid and diluting as required. All the solutions were prepared with deionized water (Milli-Q and Direct-Q). The 3,4-*ortho*-xylendioxythiophene was synthesized in our laboratory following a published methodology for the synthesis of 3,4-alkoxythiophenes [38]. A single-compartment, three-electrode cell, equipped with a glassy carbon working electrode (geometric area = 0.07 cm^2^, BASi^®^), a Pt wire as the auxiliary electrode, and an Ag/AgCl reference electrode (BASi^®^), was used. All electrochemical experiments were performed using a μAutolab type III potentiostat (Metrohm). The electropolymerization of EDOT and XDOT on the surface of glassy carbon was carried out following a reported methodology [26]. Briefly, a GC electrode was dipped in a 0.1 M LiClO_4_, 5 mM monomer ACN solution. Afterwards, the potentiodynamic polymerization was performed by sweeping between −1.2 and 1.37 V, and −0.5 V and 1.35 V vs. Ag/AgCl for EDOT and XDOT, respectively, *v* = 25 mV/s, for 10 cycles. All the electropolymerizations were performed under a N_2_ atmosphere. For all the potentiodynamic experiments, the second cycle is presented. The FTIR samples were prepared using a carbon sheet (thickness = 15 µm, width = 0.3 cm, length = 3 cm, and resistance ≈ 550 ohms) covered with a Au layer of 60 nm as a working electrode, following the above-mentioned methodology. The FTIR measurements were carried out in a FTIR Thermo Scientific Nicolet iS10 spectrophotometer. All the FTIR spectra were obtained as % of reflectance.

## Data Availability

Data are available upon request to the authors.

## Supplementary Materials

The following supporting information can be downloaded at https://www.mdpi.com/article/10.3390/molecules28020569/s1. Figure S1: SWASV of Cu^2+^ on a GC electrode; Figure S2: Potentiodynamic electropolymerization of PEDOT and PXDOT. Figure S3: FTIR spectra of PEDOT and PXDOT.

## Sample Availability

Samples of the compounds are not available from the authors.
