# Supplementary material for: Electrochemical and Spectroscopic (FTIR) Evidence of Conducting Polymer-Cu Ions Interaction
Source: Molecules. 2023 Jan 6;28(2):569. doi: 10.3390/molecules28020569 (PMC9865005; doi:10.3390/molecules28020569)
Supplement: Supplementary file 1 [file molecules-28-00569-s001.zip › molecules-2087570-supplementary.pdf]

# Electrochemical and spectroscopic (FTIR) evidence of conducting polymer-Cu ions interaction

Gerardo Salinas[1,2], Bernardo A. Frontana-Uribe[1,2]*

[1] Departamento de Química Orgánica, Centro Conjunto de Investigación en Química Sustentable UAEM-UNAM, Km 14.5 Carretera Toluca-Atlacomulco, Toluca 50200, México
[2] Departamento de Química Orgánica, Instituto de Química, Universidad Nacional Autónoma de México, Ciudad Universitaria, Ciudad de México 04510, México

*Corresponding authors: bafrontu@unam.mx

Table of contents

**Figure S1.** SWASV of 0.5 ppm $Cu^{2+}$ in a 0.1 M $LiClO_4$ acetate buffer (pH 4), obtained with a GC electrode. For all the experiments, $E_d = -1.0$ V, $t_d = 60$ s, $f = 15$ Hz, $\Delta Es = 4$ mV, $E_{SW} = 50$ mV as analytical parameters. The accumulation of the metal ion was carried out under stirring.

**Figure S2.** Potentiodynamic electropolymerization of PEDOT (bottom) and PXDOT (top) obtained in an Au/carbon sheet electrode dipped in a 0.1 M $LiClO_4$/ACN solution, $v = 25$ mV/s, monomer concentration 20 mM.

**Figure S3.** FTIR spectra of (a) PEDOT and (b) PXDOT films obtained before (black line) and after (green line) dipping the samples for 24 hr in a 0.1 M $CuSO_4$ solution.

**Additional Data**

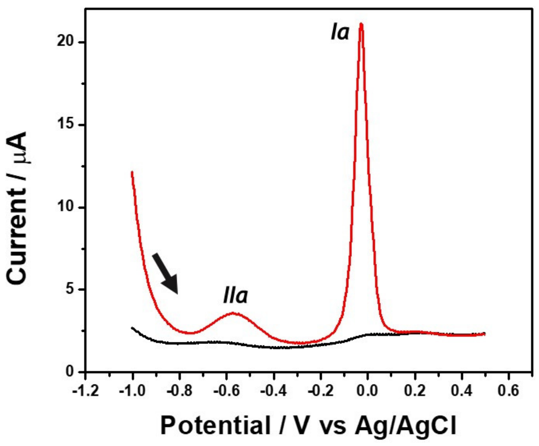


**Figure S1.** SWASV of 0.5 ppm $Cu^{2+}$ in a 0.1 M $LiClO_4$ acetate buffer (pH 4), obtained with a GC electrode. For all the experiments, $E_d = -1.0$ V, $t_d = 60$ s, $f = 15$ Hz, $\Delta Es = 4$ mV, $E_{SW} = 50$ mV as analytical parameters. The accumulation of the metal ion was carried out under stirring.

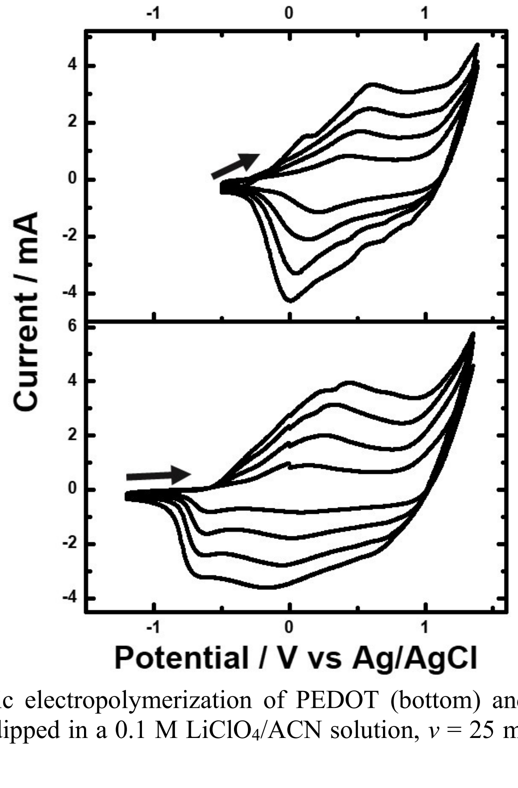


**Figure S2.** Potentiodynamic electropolymerization of PEDOT (bottom) and PXDOT (top) obtained in an Au/carbon sheet electrode dipped in a 0.1 M $LiClO_4$/ACN solution, $v$ = 25 mV/s, monomer concentration 20 mM.

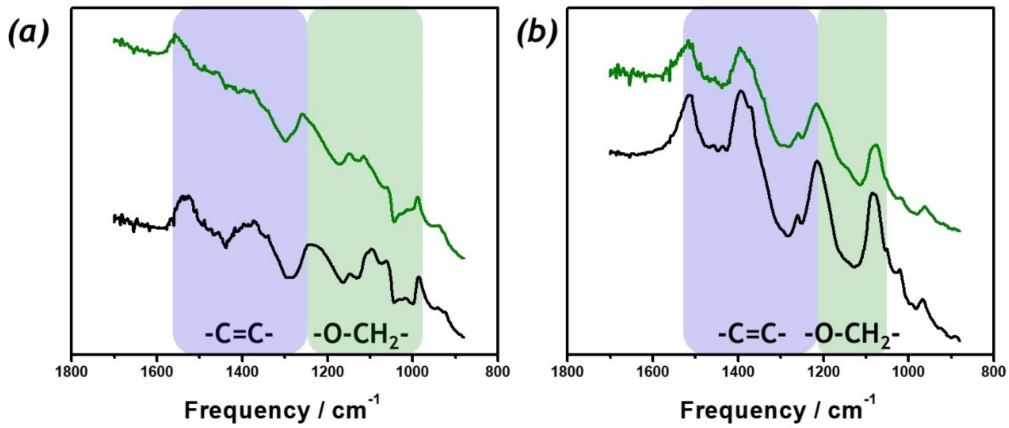


**Figure S3.** FTIR spectra of (a) PEDOT and (b) PXDOT films obtained before (black line) and after (green line) dipping the samples for 24 hr in a 0.1 M $CuSO_4$ solution.
